# Plant-Generated Artificial Small RNAs Mediated Aphid Resistance

**DOI:** 10.1371/journal.pone.0097410

**Published:** 2014-05-12

**Authors:** Hongyan Guo, Xiaoguang Song, Guiling Wang, Kun Yang, Yu Wang, Libo Niu, Xiaoying Chen, Rongxiang Fang

**Affiliations:** 1 State Key Laboratory of Plant Genomics, Institute of Microbiology, Chinese Academy of Sciences, Beijing, China; 2 National Plant Gene Research Center, Beijing, China; 3 University of Chinese Academy of Sciences, Beijing, China; 4 School of Life Science and Technology, University of Electronic Science and Technology of China, Chengdu, China; The Ohio State University, United States of America

## Abstract

**Background:**

RNA silencing is an important mechanism for regulation of endogenous gene expression and defense against genomic intruders in plants. This natural defense system was adopted to generate virus-resistant plants even before the mechanism of RNA silencing was unveiled. With the clarification of that mechanism, transgenic antiviral plants were developed that expressed artificial virus-specific hairpin RNAs (hpRNAs) or microRNAs (amiRNAs) in host plants. Previous works also showed that plant-mediated RNA silencing technology could be a practical method for constructing insect-resistant plants by expressing hpRNAs targeting essential genes of insects.

**Methodology/Principal findings:**

In this study, we chose aphid *Myzus persicae* of order Hemiptera as a target insect. To screen for aphid genes vulnerable to attack by plant-mediated RNA silencing to establish plant aphid resistance, we selected nine genes of *M. persicae* as silencing targets, and constructed their hpRNA-expressing vectors. For the acetylcholinesterase 2 coding gene (*MpAChE2*), two amiRNA-expressing vectors were also constructed. The vectors were transformed into tobacco plants (*Nicotiana tabacum* cv. Xanti). Insect challenge assays showed that most of the transgenic plants gained aphid resistance, among which those expressing hpRNAs targeting V-type proton ATPase subunit E-like (V-ATPaseE) or tubulin folding cofactor D (TBCD) genes displayed stronger aphicidal activity. The transgenic plants expressing amiRNAs targeting two different sites in the *MpAChE2* gene exhibited better aphid resistance than the plants expressing *MpAChE2*-specific hpRNA.

**Conclusions/Significance:**

Our results indicated that plant-mediated insect-RNA silencing might be an effective way to develop plants resistant to insects with piercing-sucking mouthparts, and both the selection of vulnerable target genes and the biogenetic type of the small RNAs were crucial for the effectiveness of aphid control. The expression of insect-specific amiRNA is a promising and preferable approach to engineer plants resistant to aphids and, possibly, to other plant-infesting insects.

## Introduction

The phenomenon of RNA silencing was first discovered during plant transgenic studies where it was termed co-suppression [1], [2]. Although it has various terms, post-transcriptional gene silencing (PTGS) in plants, RNA interference (RNAi) in animals and quelling in fungi, RNA silencing is based on a highly conserved mechanistic frame [1]–[3], and is a natural regulatory mechanism acting against genomic intruders and modulating endogenous gene expression in eukaryotes [4]–[7]. Because RNA silencing can be exploited to regulate gene expression through knock-down of the nucleotide sequence-matched target transcripts, it has become an effective reverse genetics approach in functional genomics and a powerful tool to develop transgenic plants that have enhanced resistance against diseases caused by e.g. virus pathogens or insect pests [8]–[13].

Earlier works on virus-resistant transgenic plants were based on pathogen-derived resistance (PDR) which was mediated by expression of viral sequences coding for, e.g. coat protein or RNA-dependent RNA-polymerase of RNA viruses or replication-associated protein of DNA viruses [14]–[16]. Even though the underlying mechanisms are not fully understood, both protein- and RNA-mediated interferences are thought to co-exist, and these strategies have been extensively used as tools to engineer virus-resistant transgenic plants [17]. As the RNA silencing mechanisms were demonstrated and studied intensively, many studies focused on the application of these RNA-mediated strategies to generate transgenic plants that are resistant to virus and fungal pathogens and insects [11], [18]–[20]. Among them, sense or antisense RNA, hairpin RNA or dsRNA, and artificial microRNA (amiRNA) were harnessed to induce RNAi in transgenic plants [21]. The works done by Mao *et al.* and Baum *et al.* showed the potential of RNAi induced by plant-expressed dsRNAs as an effective defense approach against insects, such as coleopteran and lepidopteran pests [18], [19], [22]. Recently, the plant-mediated RNAi approach was used to knockdown hemipteran genes, and the results showed a promising potential for this method in the control of sap-sucking pests [12], [23].

Aphids (family *Aphidoidea*, order *Hemiptera*) are considered economically important crop pests owing to their destruction of plants and transmission of various plant viruses. The methods of using RNAi to regulate aphid gene expression include artificial feeding of dsRNA, microinjection of dsRNA or small interfering RNA (siRNA), and transgenic plant-mediated RNAi which is a feasible way in the practice of aphid control [12], [24]–[27]. Unlike cotton bollworm (*Helicoverpa armigera*) and western corn rootworm (*Diabrotica virgifera virgifera* LeConte), which have chewing-type mouthparts, aphids, with piercing-sucking mouthparts, can only suck the sap from the plant phloem. This raised an issue that the amount of *in planta* expressed dsRNAs sucked by aphids in the sap-sucking may be too low to trigger the RNA silencing.

To test the feasibility of transgenic plant-mediated RNAi in resistance against aphids, we chose *Myzus persicae*, which can colonize diverse plant species and could be reared on tobacco or Arabidopsis plants in the greenhouse, as a target. Based on the sequence information in GenBank, nine *M. persicae* genes were chosen to be subjected to RNAi. The intron-spliced hairpin RNA (hpRNA)-expressing plant vectors were constructed. Besides, for the gene *MpAChE2*, which encodes a key enzyme in the insect central nervous system, two amiRNA-expressing vectors were also constructed. The plant expression vectors were transformed into *Nicotiana tabacum* cv. Xanti. Insect challenge assays showed that, compared with the untransformed plants, most of the transgenic plants displayed aphid resistance, and transgenic plants expressing V-ATPaseE or TBCD hpRNAs showed a relatively higher level of resistance. It is worthy to note that *MpAChE2* amiRNA (amiR-AChE2)-expressing transgenic plants showed better aphid resistance than plants expressing *MpAChE2* hpRNAs (hpAChE2), and the aphids reared on the amiR-AChE2 expressing lines showed a more evident reduction in the target gene transcript level. In summary, the plant-mediated RNAi approach is a practical way to develop aphid-resistant plants, and selection of vulnerable aphid genes and the biogenetic type of the small RNA used in RNAi are two important considerations. Host expression of insect-specific amiRNAs is a promising new approach to engineer aphid-resistant, or even other insect-resistant, plants.

## Results

### Selection and Cloning of *M. persicae* Target Genes

Based on the results of a dsRNA-feeding assay in western corn rootworm conducted by Baum *et al.* [18], eight aphid genes encoding proteins with essential functions were selected as our RNAi targets (Table 1). Since there is no sequence information available for the *M. persicae* genes and the genome sequence of the pea aphid *Acyrthosiphon pisum* is published [28], [29], we referred the corresponding sequences of *A. pisum* to design the PCR primers. The 300 to 500 bp DNA fragments of the target genes were obtained by PCR from the cDNAs of *M. persicae* with specific primers. The gene sequences are shown in Figure S1 and their intron-spliced hpRNA expression vectors were constructed. Besides, one more gene, *MpAChE2* encoding acetylcholinesterase 2 which plays vital roles in insect growth and development, was chosen to construct a hpRNA expression vector and two amiRNA expression vectors (Table S1, Figure S2).

**Table 1:** Genes from *Acyrthosiphon pisum* chosen for RNA interference in *Myzus persicae*.

Candidate target Gene	Abbreviate	GenBank Accession number	NCBI Accession number
V-type proton ATPase subunit E-like	A-V-ATPaseE	CN582351	NM_001162178
RR1 cuticle protein 1 (cprr1-1)	B-CPRR1	CN749119	NM_001162314
40S ribosomal protein S5-like isoform-1	C-Rps5	CN749546	XM_001949078
SWI/SNF-related matrix-associated actin-dependent regulator of chromatin subfamily D member 1-like	D-SMARCD1	CN582484	XM_001945531
tubulin folding cofactor D (TBCD)	E-TBCD	DY226108	XM_001950755
coatomer subunit delta-like	F-delta-COP	CN754179	XM_001944394
ribosomal protein S14 (Rps14)	J-Rps14	CF587619	NM_001162581
mediator complex subunit 31 (Med31)	K-Med31	DV744955	NM_001162703
acetylcholinesterase 2	I-AChE2	AY147797*	XM_001948618
acetylcholinesterase 2	G/amiR-AChE2-1		
acetylcholinesterase 2	H/amiR-AChE2-2		

*AY147797 is the GenBank accession number of *M. persicae* acetylcholinesterase 2.

### Characterization of Transgenic Tobacco Plants

Transgenic tobacco lines were generated by *Agrobacterium tumefaciens*-mediated transformation, and identified by PCR. The seeds of T0 transgenic plants were harvested and screened by hygromycin B selection and the hygromycin resistant T1 plants were further tested by PCR. All the confirmed T1 transgenic plants were self-fertilized, and the homozygous T1 plants which were identified by segregation ratio of their T2 plants on hygromycin selection were maintained for further testing. Three independent transgenic lines of each of the hpRNA- and amiRNA-expressing plants were chosen for insect-resistance assay, none of which displayed obvious phenotypic aberrations.

### Insect Resistance of Transgenic Tobacco

**Figure 1:**
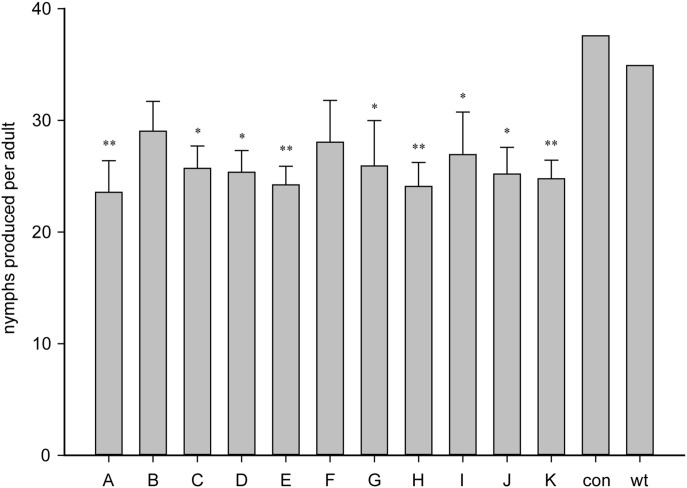
Transgenic tobacco lines expressing artificial hairpin RNAs or microRNAs have effects on aphid fecundity. A–F and I–K represent transgenic plants expressing hpRNA corresponding to genes V-ATPaseE, CPRR1, Rps5, SMARCD1, TBCD, and delta-COP, and AChE2, Rps14 and Med31, respectively, of *Myzus persicae*, while G and H represent transgenic plants expressing amiRNA targeting AChE2. The control (con) transgenic plants contained an empty vector. Bars represent the means (±SE) of three lines of each genotype. The experiment was repeated at least twice with similar results. Asterisks represent significant differences between the transgenic and the wild type (wt) as determined by Student’s *t*-test (An asterisk denotes p<0.05, and a double asterisk indicates p<0.01). In each test, the fecundity of each construct was the average value from three independent lines, and the fecundity of each line was collected from three plants.

To investigate if plant-mediated silencing of the candidate target genes can affect the fecundity of *M. persicae*, the following challenge assay was conducted. Three independent transgenic lines for each construct were selected, three seedlings of each independent line were tested and the experiment was repeated at least twice. Compared with untransformed tobacco plants and empty vector-transgenic plants, most of the transgenic plant lines showed some degrees of aphid resistance (Figure 1). As shown in Figure 1, columns A and E, representing the V-ATPaseE and TBCD hpRNA-expressing transgenic plants, respectively, showed relatively higher aphid resistance, which represented 32% and 30% reduction of nymphs produced by each adult, respectively. Interestingly, transgenic plants expressing amiR-AChE2 that targeted 1311–1331 nt and 1990–2010 nt of the ORF showed better aphid resistance than those expressing hpAChE2. This was especially evident when the target site was located toward the 3′ end of the mRNA, as in amiR-AChE2-2 which targeted 1990–2010 nt of the ORF (Figure 1-H, Figure S2).

### Expression Profiles of *MpAChE2*

**Figure 2:**
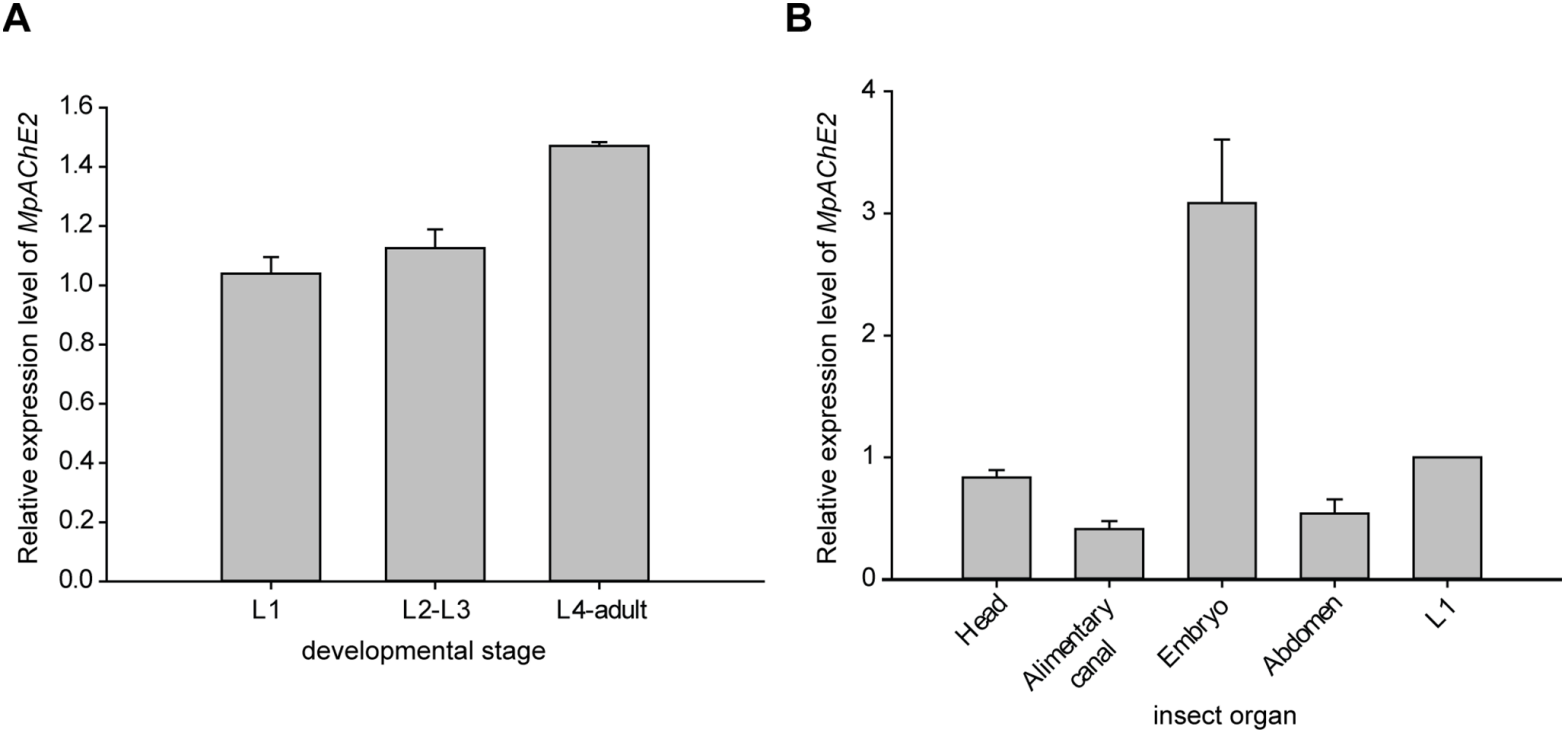
Expression profiles of the acetylcholinesterase 2 coding gene (*MpAChE2*). (A) qRT-PCR analysis of the *MpAChE2* expression at different developmental stages of *Myzus persicae*. (B) Relative expression level of *MpAChE2* in adult aphid organs with the expression level in the whole body of L1 instar nymph as one (L1). The housekeeping gene, *α-actin*, was used as an internal control. The data represent the means (±SE) of three replicates.

The full length cDNA of *MpAChE2* was cloned using Rapid amplification of 3′ cDNA ends (3′ RACE) and PCR (Figure S2). Its expression at various developmental stages was investigated by quantitative RT-PCR (qRT-PCR). The developmental expression pattern revealed that the *MpAChE2* transcript was present in all developmental stages of *M. persicae* and increased from the L1 instar to the adult stage (Figure 2A). To explore the organ distribution, qRT-PCR was used to quantify the *MpAChE2* transcipts from the cDNA of different adult aphid organs. The results showed that *MpAChE2* expressed in every aphid body organ tested and was more abundant in the embryos (Figure 2B).

### Effects of the hpAChE2 and amiR-AChE2 Transgenic Tobacco on Insect Target Gene

**Figure 3:**
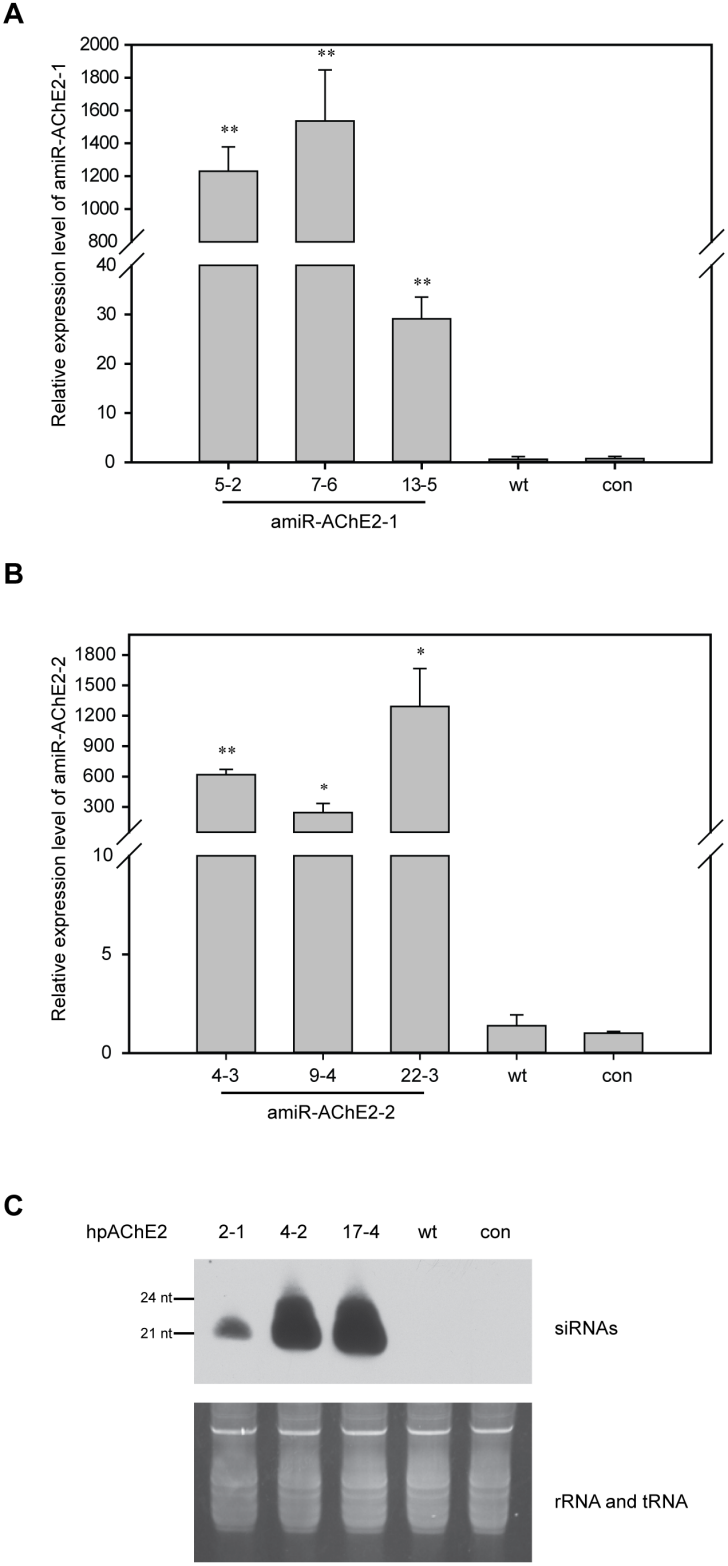
Expression levels of artificial microRNAs and small interfering RNAs, targeting the *MpAChE2* gene of *Myzus persicae*. Relative expression levels of amiR-AChE2-1 (A) or amiR-AChE2-2 (B) in tobacco plants analyzed by qRT-PCR. The expression levels of amiRNAs were relative to the expression of tobacco 5.8S rRNA and normalized. The data represent the means (±SE) of three biological replicates. Student’s *t*-test was used to determine significant differences in transgenic plants compared to wild type plants (An asterisk denotes p<0.01, and a double asterisk indicates p<0.001). (C) Northern blot detection of AChE2 siRNAs in plants. rRNA/tRNA stained with ethidium bromide was used as a loading control.

**Figure 4:**
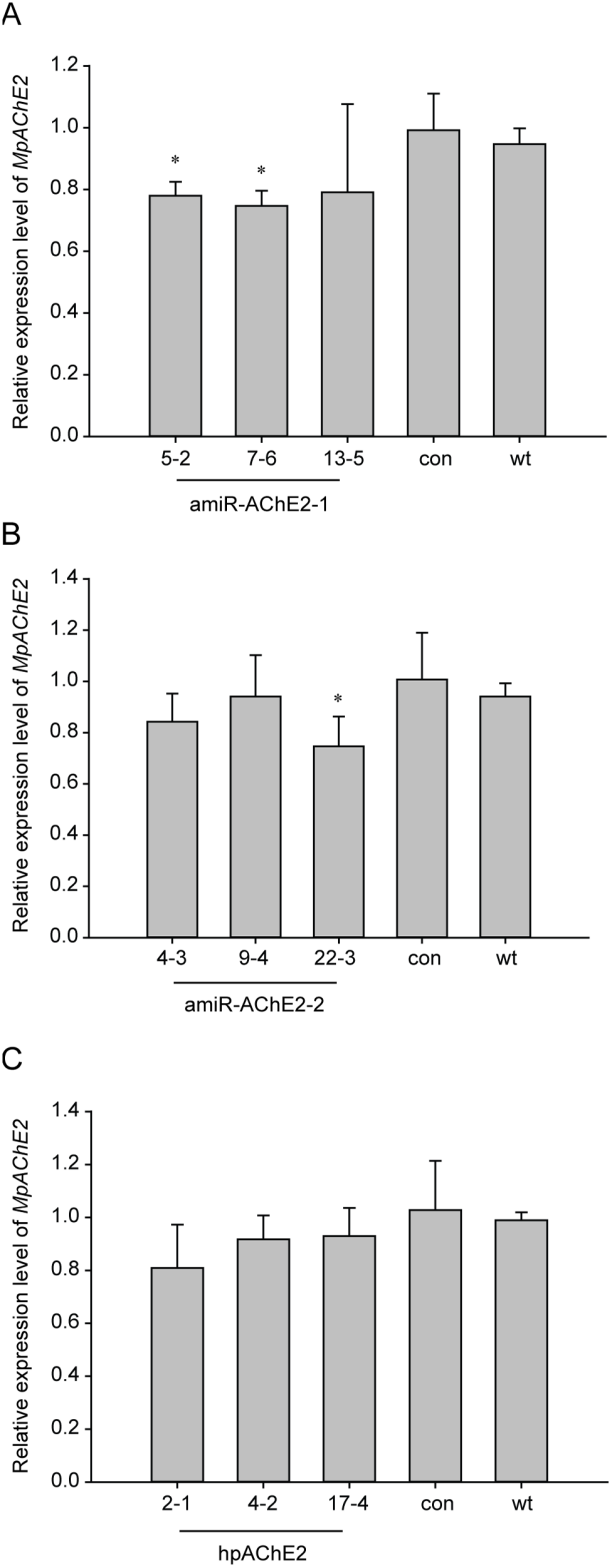
Knock-down of *MpAChE2* expression by plant-mediated RNAi. Transgenic tobacco lines expressing amiR-AChE2 or hpAChE2 down-regulated the target gene expression compared to empty vector-transgenic plants (con) and wild type (wt). The data represent the means (±SE) of three biological replicates. Student’s *t*-test was used to determine significant differences in aphid fed on transgenic plants compared to aphid fed on wild type plants (An asterisk denotes p<0.05).

To confirm that small RNAs endow insect resistance to transgenic plants, the transgenic small RNA expression levels of each of the three independent lines of hpAChE2, amiR-AChE2-1 and amiR-AChE2-2 transgenic plants were determined by northern blot or qRT-PCR (Figure 3). The results showed that the small RNAs derived from both hpRNA and pre-amiRNA existed in the transgenic plants. The siRNAs corresponding to *MpAChE2* were similarly abundant in lines 4-2 and 17-4, less abundant in line 2-1 (Figure 3C), while the amiR-AChE2-1 and amiR-AChE2-2 were more abundant in lines 7-6 and 22-3, respectively, than in other lines (Figure 3A, B). Furthermore, the target gene expression levels in feeding aphids were assessed by qRT-PCR. The results showed that the expression levels of the target gene decreased in aphids fed on transgenic plants compared to those fed on control plants (Figure 4). The decrease was more significant in aphids feeding on amiR-AChE2-expressing plants than that in aphids feeding on hpAChE2-expressing plants. Particularly, *MpAChE2* was more down-regulated on amiR-AChE2-1 line 7-6 and amiR-AChE2-2 line 22-3 (Figure 4A, B), in inverse relation to the abundance of amiR-AChE2 in transgenic plants. These results indicated that the plant-expressed small RNAs, both hpRNA and amiRNA, could cause RNAi in the aphid and the latter one displayed more effective and predictable insect resistance.

## Discussion

RNAi was first discovered in *Caenorhabditis elegans* [2]. The expression of long dsRNA in *C. elegans* results in an efficient sequence-specific inhibition of gene expression. A follow-up study revealed that the dsRNA could be provided to *C. elegans* by feeding or injection to knock out a specific endogenous gene [30]. The injection of dsRNA into insects to down-regulate target genes has been widely used as a biological research tool [25], [31], [32]. Mao *et al.* and Baum *et al.* demonstrated that dsRNA expressed *in planta* could result in the silencing of the corresponding gene in chewing herbivores, and it showed the potential of RNAi as a new way to generate insect-resistant crops [18], [19]. It was indicated that both the selection of target gene and the concentration of the dsRNA are important for plant resistance against insect [18]. Additionally, when aphids fed on *N. benthamiana* leaf discs transiently producing dsRNA corresponding to *Rack1* and *MpC002* genes and on *Arabidopsis thaliana* plants stably producing these dsRNAs, the expression levels of the *MpC002* and *Rack-1* genes in aphids were knocked down by up to 60%, and aphids produced less progeny [12]. Obviously, RNAi is a feasible method for insect-resistance in plant, and the selection of the target gene is crucial.

To search for more candidate genes, and create a more effective method, nine putative essential aphid genes were selected as RNAi targets. Transgenic tobacco lines that expressed the corresponding hpRNA were generated. Because aphids reproduce asexually, based on our observations, a first instar nymph will give birth in seven to eight days after feeding on tobacco plants and will continue to give birth for the following eight days. When we performed insect challenge assays, the total number of aphids was counted on the fourteenth day after the insects were fed on the plants. Thus, besides the original five adult aphids, there are only sibling aphids in the test tubes. Our data showed that transgenic plants expressing hpRNAs of essential insect genes presented degrees of insect resistance and that different target genes showed different effects, which agrees with previous published data [18].

miRNAs are another class of RNA silencing effectors that function as negative regulators of gene expression in plants. Previous work indicated that the miR171/miR171* sequences in the stem of the Arabidopsis pre-miR171a could be replaced by a 21-bp DNA fragment (targeting the CMV 2b gene) containing two mismatches, imitating the mismatches between the miR171 and miR171* sequences. The precursor of amiRNA can be transcribed and processed correctly in transgenic tobacco plants, and the amiRNA transgenic plants had better virus resistance than the short hairpin RNA transgenic plants [4]. To compare the efficacy of hpRNA and amiRNA in generating insect resistance, one hpRNA-expressing vector and two amiRNA-expressing vectors aimed at the aphid *MpAChE2* gene were constructed. Acetylcholinesterase is a hydrolase that hydrolyzes the neurotransmitter acetylcholine. It is the primary target of the nerve agent sarin and organophosphates that interfere with the acetylcholinesterase, disrupting nerve impulses and killing or disabling the insects. Our results showed that *MpAChE2* expressed in every aphid body organ tested and was more abundant in aphid embryos (Figure 2B). Thus, it should be important for the growth, behavior and reproduction of insects. The Arabidopsis pre-miR171a was used as framework for amiRNA expression by replacing the native stem fragment of pre-miR171a with 21-bp targeting sequences. The two targeting sequences and their locations in the gene *MpAChE2* are shown in Figure S2.

A test of the expression levels of *MpAChE2* in aphids showed that the *MpAChE2* transcript levels decreased in aphids feeding on hpRNA or amiRNA transgenic plants compared with the transcript levels in insects feeding on control plants (Figure 4). The decrease was more significant in aphids feeding on amiRNA transgenic plants than in aphids feeding on hpAChE2 transgenic plants. The reason for this difference could be complicated but the higher expression level, stem stability of the amiRNA and the specificity to the target may be contributing factors. This result is in accordance with the insect resistance test, where the amiR-AChE2-2 transgenic plants showed better insect resistance. Aphids feeding on the amiR-AChE2-2 transgenic plants had slightly higher *MpAChE2* transcript levels than those feeding on amiR-AChE2-1. Individual differences might cause this divergence since the target gene’s transcript levels were only tested in a very small portion of the aphids.

In summary, our results showed that small RNAs produced by transgenic plants can cause RNAi in insects feeding on the transgenic plants, whether the small RNAs are processed from hpRNA or pre-amiRNA. However, the target genes selection is important. Among the genes selected in this study, the V-ATPaseE and TBCD proved to be the most vulnerable target genes. For the same target gene, e.g. *MpAChE2*, transgenic plants expressing amiRNA appeared slightly better in producing insect resistance, which implies that the amiRNA strategy might be a better way to engineer aphid-resistant transgenic plants. Not only did the transgenic plants constructed by this method display better insect resistance but they were also more specific and contained shorter sequences of the target gene than hpRNA containing plants. This may reduced the off-target effects, leading to more precise and biosafer insect-resistant transgenic plants.

## Materials and Methods

### Insect Rearing

The aphid lineage used in this study came from the tobacco (*N. tabacum* cv. Xanthi) plants in our greenhouse. A single aphid was reared on sterile tobacco plant grown in tissue culture tubes. The progeny aphids were maintained on tobacco in controlled environmental conditions at 25°C under 16 h light and 8 h dark from generation to generation, and this aphid lineage was identified as belonging to *M. persicae* by the Institute of Zoology, Chinese Academy of Sciences.

### Rapid Amplification of *MpAChE2* cDNA End

Total RNA was extracted from adult *M. persicae* using TRIzol reagent (Invitrogen, Carlsbad, CA, USA). 3′ RACE was performed with the 3′-Full RACE Core Set (TAKARA, Dalian, China) according to the manufacturer’s instructions using primers AChE2-outer and AChE2-inner (Table S1). These two primers were designed according to the known partial CDS of *MpAChE2* mRNA under the accession number AY147797. PCR products were purified, ligated to the pGEM-T Vectors (Promega, Madison, WI, USA) and sequenced. The full-length *MpAChE2* cDNA sequence was obtained by linking the above two sequences.

### Cloning and Vector Construction

Sequence information for the target genes came from genome sequence of pea aphid *A. pisum* in GenBank (Table 1). Sequence-dependent primers were synthesized and used to amplify gene sequences from the cDNAs of *M. persicae*. For each gene fragment, two pairs of primers were synthesized, and different restriction sites were incorporated into the primers (Table S1). The gene fragments were amplified and ligated into the pGEM-T vectors and each construct was confirmed by sequencing (Figure S1). The construction of plant hpRNA expression vectors were based on the pIPK plasmid from our laboratory [33]. pIPK was modified based on pCAMBIA-HAN derived from pCAMBIA1300 and pHANNIBAL, which contains the *Cauliflower mosaic virus* (CaMV) 35S promoter, an intron from the pyruvate orthophosphate dikinase gene (*pdk*) and the transcription terminator of the octopine synthase gene (*ocs*) [34]. Restriction sites were available on both sides of the intron in the pCAMBIA-HAN vector (Figure S3A), and the plasmid contains a hygromycin B resistance gene for transgenic plant selection. The PCR-amplified fragments encoding the sense RNA of the target genes were digested with *Eco*RI and *Kpn*I and gel purified. They were then ligated into the pIPK plasmid and digested with the same restriction enzymes. After transformation into the DH5α *Escherichia coli* strain and confirmation by sequencing, the resulting plasmids were digested with *Bam*HI and *Xba*I for antisense target gene fragment cloning. The target gene fragments with *Bam*HI and *Xba*I sites were digested with the enzymes, gel purified and ligated into the appropriate prepared plasmids. All the resulting plasmids were confirmed by sequencing and transformed into *A. tumefaciens* EHA 105.

The plant amiRNA expression vectors were generated as described by Qu *et al*. [4]. The CaMV 35S promoter was chosen to direct the expression of amiRNA from the *A. thaliana* pre-miR171a backbone. Two pairs of primers were synthesized, corresponding to the 5′ (sense) and 3′ (antisense) portions of pre-miR171a, with each harboring a 21-nt *MpAChE2*-specific sequence (underlined) which targeted a corresponding site on the *MpAChE2* gene (Table S1, Figure S2). Each pair of primers were annealed to the DNA template present in plasmid pGEMT-premiR171a and extended by PCR. The resulting amplified products, pre-miR171a/ AChE2-1 and pre-miR171a/ AChE2-2, were chimeric DNAs (miR-AChE2prec1 and miR-AChE2prec2) that were cloned into the plasmid pBI221 via the *Bam*HI and *Sac*I sites to generate pBI-35S-miR-AChE2prec1 and 2, respectively. The 35S-miR-AChE2prec1 and 2 expression cassettes were transferred into pCAMBIA1300 to generate p35S-miR-AChEprec1 and 2 plasmids, denoted as amiR-AChE2-1 and amiR-AChE2-2, respectively (Figure S3B).

### Generation of Transgenic Plants

Tobacco (*N. tabacum* cv. Xanthi) plants were transformed by the *Agrobacterium*-mediated leaf disc method with some modifications [35]. pCAMBIA1300-221, a derivative of pCAMBIA1300 and pBI221, was transformed as a control. The primary transformants (T0) were first selected by PCR for the presence of the transgene. PCR-positive plants were allowed to self-pollinate, and T1 seeds were selected on germination medium containing hygromycin B (50 mg/L). The hygromycin B resistant plants were allowed to self-fertilize and seeds of the homozygous plants that could not segregate hygromycin B-sensitive plants were harvested for the insect challenge experiment.

### Quantifying amiRNA Expression by Real-time PCR

AmiRNA expression was quantified by real-time PCR as described previously with some modifications [36]. Total RNA was isolated from tobacco plants using TRIzol reagent and treated with TURBO DNA-free kit (Ambion, Austin, TX, USA). The treated total RNA (1 µg) was polyadenylated using a Poly(A) Tailing Kit (Ambion). Poly(A)-tailed RNA was recovered by phenol/chloroform extraction, ethanol precipitation and reverse-transcribed by SuperScript III reverse transcriptase (Invitrogen) using 50 pmol of poly(T) adapter (Table S1) as primers. *N. tabacum* cv. Xanthi 5.8S ribosomal RNA (rRNA) was selected as the internal standard. To amplify amiRNA from the reverse transcribed cDNAs, we used the amiRNA sequence as the forward primer (Table S1). qRT-PCR was conducted with a Bio-Rad CFX96 real-time PCR detection system (Bio-Rad, Hercules, CA, USA.) and the Thunderbird SYBR qPCR Mix (Toyobo, Osaka, Japan), and analyzed by the 2^−ΔΔCt^ method.

### Northern Bolt Analysis

Total RNAs were extracted from four-week-old tobacco plants using TRIzol according to the manufacturer’s instructions and 30 µg samples were separated on 15% polyacrylamide gels containing 7 M urea. They were then transferred to Amersham Hybond NX membranes (GE Healthcare, Buckinghamshire, UK). The gel was stained with ethidium bromide before transfer to confirm equal loading. N-(3-Dimethylaminopropyl)-N′-ethylcarbodiimide hydrochloride (EDC) mediated cross-linking was used as previously described [37]. The probes for detecting *MpAChE2* siRNAs were labeled with biotin-11-dUTP using the Prime-a-Gene Labeling System (Promega). The membrane was detected with a Chemiluminescent Nucleic Acid Detection Module (Pierce, Rockford, USA) according to the manufacturer’s instructions.

### Aphid Challenge Assay

To screen for insect resistant transgenic tobacco plants, the homozygous seeds of transgenic plants were grown in Petri dishes (100 mm×10 mm) containing 25 ml of MS medium with 50 mg/L of hygromycin B. Young tobacco seedlings, 10 days post-germination, were transferred into culture tubes (60 mm×125 mm), with each tube containing only one plant. At the five-leaf stage, the seedlings were inoculated with three adult aphids using a writing brush. After five days, all adults were removed and five approximately two-day-old nymphs remained. Fourteen days later the total number of the aphids on each seedling was counted, and that number was recorded as the fecundity of the original five nymphs. Three independent lines of each construct were chosen for the assay, and three plants per line were inoculated. Untransformed tobacco plants and empty vector transgenic plants were inoculated in parallel as negative controls.

### *MpAChE2* Expression Analysis in Aphid by qRT-PCR

qRT-PCR was performed to analyze the expression level of *MpAChE2* at different stages and organs in *M. persicae*. To check the *MpAChE2* expression level in aphids feeding on transgenic plants, we chose three adults from the tobacco plants on the fourteenth day of the aphid challenge assay. Total RNA was extracted using TRIzol reagent and treated with TURBO DNA-free kit. The RNA was then used for the synthesis of cDNA by SuperScript III Reverse Transcriptase using oligo(dT)_18_ as primers according to the manufacturer’s instructions. Gene specific primers were designed to amplify a 175-bp fragment of *MpAChE2* cDNA. *Actin* was used as an internal control. qRT-PCR was conducted as described above.

## Supporting Information

Figure S1 **Nucleotide sequences of** ***Myzus persicae*** **target genes.**
(PDF)

Figure S2 **Complete coding sequence of acetylcholinesterase 2 (** ***MpAChE2*** **) in** ***Myzus persicae*** **.** The start codon (ATG), the stop codon (TTA) and artificial microRNAs (amiRNAs) targets are highlighted in the figure.
(PDF)

Figure S3 **Structures of the constructs for expressing artificial hairpin RNAs (A) and microRNAs (B) in plants.**
(TIF)

Tables S1 **List of primers used.**
(DOC)
