# Supplementary material for: Plant-Generated Artificial Small RNAs Mediated Aphid Resistance
Source: PLoS One. 2014 May 12;9(5):e97410. doi: 10.1371/journal.pone.0097410 (PMC4018293; doi:10.1371/journal.pone.0097410)
Supplement: Figure S1 — Nucleotide sequences of Myzus persicae target genes. (PDF) [file pone.0097410.s001.pdf]

Figure S1. Nucleotide sequences of *Myzus persicae* target genes.

V-type proton ATPase subunit E-like (312 bp)
5′-AGCGACTCAAGATCATGGAGTATTTTGAGCGCAAAGAGAAACAAGTGG
AGTTACAGAAAAAAATTCAATCATCTAATATGTTAAATCAAGCACGTCTT
AAGACATTAAAAGTGCGAGAAGATCATGTCAGTGATGTATTAGATGAAGC
AAGGAAGCGTCTGGTCAAAGTGACAAATAACCCTGATTTGTACAGAGAAG
TTCTTAGAAAATTAATATTGCAAGCAATCTTACAGTTGTTGGAGAAAAAT
GTTACATTACGTGTTCGTGAAGTTGATTTATCAGTTGTGGAAGAATTAGTT
GAAGAAGTGGCAG-3′

RR1 cuticle protein 1 (362 bp)
5′-CGTCGTTGCTGTTAGCTGTCGTCGCAGTCAGCGCCTACCCCGCTGGACA
GAGCCCGGAATCCAGAGCCGTCATCTTGGTCCAAGATTCAGCACCCAGCG
CCGACGGATCATTGAAGAACAACTTCCAAACCGATAACGGAATCAAACAA
GAAGAAGTCAGGTACTTGAAGGCTGGCCCAGAAGGACCCGTTTCAGTTGT
CCAAGGAGCAGTGTCTTACGTGGCCCCAGACGGCCAGACCATCCAAACCG
GCTACGTCGCCGACGAGAACGGTTACCAGCCGTACGGCGCTCACTTGCCC
ACTCCACCAGCAATCCCATTCGAGATCCAAGAGTCACTCAGGTACCTCGCC
TCTCTGCCCAGCAC-3′

40S ribosomal protein S5-like isoform-1 (395 bp)
5′-GGGGAGATGTGGGAGCTACAATGTAGATATTGCTCCAACACAAATTGCA
GCTGAATTACCAGAAGTGAAATTATTTGGCAGATGGAGTTGTGATGATGTA
GAAGTTTCTGATATGTCTTTGCAAGATTACATTGCTGTCAAAGAAAAGTACG
CCAAATATGTTCCTCACTCTGCTGGTCGTTATGCTGCTAAACGTTTCCGTAA
GGCTCAATGCCCCATTGTTGAACGTTTGACCAACTCTTTGATGATGCACGG
ACGTAATAACGGAAAGAAACTGATGGCCGTTCGCATTGTTAAACACGCTTT
TGAAATTATCTACCTGTTATCTGGTGAAAATCCATTGCAAGTGCTTGTAAAA
GCCATCATTAACAGTGGACCCAGAGAGACTCACAC-3′

SWI/SNF-related matrix-associated actin-dependent regulator of chromatin subfamily D member 1-like (499 bp)
5′-GACCCGCCGATAATCGAAATCCACCGCCTATGGTCAAAGGTGATTTTCCT
CACAACATACAAGTTCAAAATACCAGTATTGGAATGAGCAAAAAAAAGAA

AAAATTGGCGGATAAAATATTGCCCCAAAAAGTAAGAGATCTTGTTCCAGA
ATCACAAGCATACATGGATCTTTTGGCATTTGAAAGAAAATTAGATTCAACA
ATCATGAGAAAACGATTAGACATACAAGAAGCATTAAAACGTCCAATGAAA
CAAAAACGTAAATTGCGTATATTTATATCAAATACATTTTATCCGGCAAAAGA
ACCTGGAGAAAATGAAGAAGGAAGTGTTGCTTCTTGGGAATTAAGAGTTG
AAGGACGTTTACTAGAAGATTCTAAAAATGAGCCAAATAAGATAAAAAGAA
AATTTTCTTCTTTTTTTAAATCACTGGTCATTGAACTTGATAAAGATTTATAT
GGGCCTGATAATCATTTGGTTGAATGGCATCGGACCGCA-3′

tubulin folding cofactor D (489 bp)
5′-TGGCATGGAGGATGTTTAGCACTTGCTGAATTGGGCCGCCGTGGTTTACT
TCTCAGTGAAAATTTACCTCTTGTTGTACCTGCTGTTATAAAAGCTTTGGTG
TATGATGAACCTAAGGGGTATACATCCGTAGGATCACATATTAGAGATGCAG
CTTGTTATGTTTGTTGGTCTTTTGCAAGAGCATTTAGTTCTGATGACATTCA
GCCGTATGTTGAAGAAATCGCTGGCGCGTTATTAGCAGTTGCTTGTTATGAC
AGAGAGTTGACATGCCGTAGAGCAGCTTCAGCAGCATTTCAAGAGAATGTT
GGACGTCAAGGTAAATTTCCACATGGAATTGACATTGTGACAGCAGCTGAT
TATTTTTCAGTTGGTATGCGTAATAATGCTTATTTAGAAGTCAGCATTTTTATA
GCACAATATAAAGAATATCATAAACTTCTGATCAAGCATCTGTTAGAAAAGA
AAATAGTACATTGGGACACATCA-3′

coatomer subunit delta-like (448 bp)
5′-TTAACACTCTCCCTATATCCAAGGGCTACAATTTCAACAAAAGCAAATATT
AAATCAAAAGATTTTTTTGATACTTCACTCTCATCGACTAACTTGCAACAAT
CCAGGACTACTTTTGAAAACAATCTTAATGTTTCCAAATCTTCTAATATGTTA
CTGGTCTTTGTAGTAATCAATAACATGTAGAGTTTTTCCAATGGCTGGTATAC
ATATCGTACAGAATCTGTCTCAACAAATGTATGTTGAATATGACGTTTTTCTG
TCGACATCAGCTTAGGAAAAGCAGCCAGTAGACCCTCAATTCGCGCTTTAG
TCATTTCAACAAACTGTCGAGAGACAATGGTTTTTCCTGTTTTTGTGCATAC
GGCTGAAGCCAACAGCACCATTTTGGCAGTGAGTTTGTGCAAACTTGTAA
ACGTACACTCAGTCGATAATACAGTTCTTACGG-3′

ribosomal protein S14 (429 bp)
5′-AAGGAAGAGCCAGTAGTCCAACTGGGACCACAAATTCAAGAAGGTGAA
TTGGTTTATGGCGTTGCTCACATTTTTGCTAGCTTCAATGATACATTCGTGCA

TGTCACAGATTTATCTGGAAGAGAAACAATTGCTAGAGTTACTGGTGGCAT
GAAAGTAAAAGCTGACAGAGATGAAGCTTCTCCTTATGCTGCTATGTTGGC
TGCCCAAGATGTTGCTGAAAAATGTAAATTATTGGGTATCACAGCTTTGCAT
ATTAAATTGAGGGCTACTGGTGGAAACAAGACAAAAACACCTGGTCCAGG
AGCACAATCTGCATTGCGTGCTCTAGCTCGTTCAAGTATGAAAATTGGCAG
AATTGAAGATGTAACACCAATTCCCTCAGATTCTACACGTAGGAAGGGTGG
TCGCAGAGGTAGACGTTTGTA-3′

mediator complex subunit 31 (387 bp)
5′-GCTTATACCTATGACGTTTCACCGAGTGGCGTCATGACATGATTGTTTGA
GTTGCAAATCTATTGTATAAACAAGTCGACAATTTACTTAAATAACACAAAA
TCTAATGAAGAATTAACCAAACCAAAATGGCTAACAAAGGTGGTCCTGAA
ACTGAAGAACAAACCAGATTAAGGTTTCAAGTAGAACTTGAATTTGTTCAA
TGCTTAGCTAATCCAAATTATTTAAACTTTTTAGCTCAACGTGGATACTTTAA
GGATCAGTCTTTTATTAATTATTTAAAATATTTACTATACTGGAAAGAGCCAG
ATTATGCAAAATTTATTAAGTATCCCATGTGTTTGTACTTTTTAGATCTTTTAC
AGCATGAACCATTTAGAAAAGAAATAGCTACTGCTATTTGTTCGAAATTTAT
GATGATCAG-3′

acetylcholinesterase 2 (376 bp)
5′-GAATTCCCCGGTATCTGAAGATTGCCTCTACATTAACGTAGTAGTGCCAA
AGCCTAGACCACAAAACGCAGCAGTGATGGTATGGATTTTCGGAGGAGGA
TTTTACTCCGGGTCTGCTACTTTGGATATTTACGACCCTAAAGTACTCGTATC
GGAAGAAAATGTGATTTTGGTATCCATGCAGTACAGAGTTGCATCTTTAGGC
TTTTTATACTTTGACACTGAAGATGTTCCGGGAAACGCTGGACTTTTTGATC
AGCTAATGGCTCTACAGTGGGTACACGAAAACATTAAATTATTTGGCGGCA
ACCCAAACAACGTGACACTTTTCGGTGAGTCAGCCGGCGCCGTTTCAGTTT
CACTGCACTTACTGTCT-3′
