# Supplementary material for: Plant-Generated Artificial Small RNAs Mediated Aphid Resistance
Source: PLoS One. 2014 May 12;9(5):e97410. doi: 10.1371/journal.pone.0097410 (PMC4018293; doi:10.1371/journal.pone.0097410)
Supplement: Figure S2 — Complete coding sequence of acetylcholinesterase 2 ( MpAChE2 ) in Myzus persicae . The start codon (ATG), the stop codon (TTA) and artificial microRNAs (amiRNAs) targets are highlighted in the figure. (PDF) [file pone.0097410.s002.pdf]

Figure S2. Complete coding sequence of acetylcholinesterase 2 (*MpAChE2*) in *Myzus persicae*. The start codon (ATG), the stop codon (TTA) and artificial microRNAs (amiRNAs) targets are highlighted in the figure.

5′-tcgcgggttttttttttgaatgatttaaattagcagtggttggtcgaacattaccactgatttgtagtttgatgatcgaattcact
gggaatttgaaaatctgtctttaaaaATGgaccagtggttgctgtggtttagctacttagtagcttctacttatggactttcattg
cgccacgccaggcaccaaagcgttgggacaccaactgccgaagaaatactggaaccacagattctaattgaagacaccg
atcatgtattcagacaacgagcgtcagatatgtttgcccaagagccagaatacacagagaagcgaaaccttaatcatagac
ggaggtccgaatttagtggaaaccaggataatgatttcgaatcttctggggagacatacagtgcgtataaatcggatgatcc
gttggtaattcacacgaacaagggtaagatcagaggaatcacacaggcagcgtcgactgggaagttagttgatgcatggct
aggaataccctacgcaaaaaaaccaataggtgatcttaggtttaggcaccctcgcccaattgaccgttgggacaatacaaa
cccagagaccattcttaactgcactactccaccaaatacatgtgttcaaatattcgacacgctttttggtgattttcccggtgcta
caatgtggaatccgaattccccggtatctgaagattgcctctacattaacgtagtagtgccaaagcctagaccacaaaacgc
agcagtgatggtatggattttcggaggaggattttactccgggtctgctactttggatatttacgaccctaaagtactcgtatcg
gaagaaaatgtgattttggtatccatgcagtacagagttgcatctttaggctttttatactttgacactgaagatgttccgggaaa
cgctggactttttgatcagctaatggctctacagtgggtacacgaaaacattaaattatttggcggcaacccaaacaacgtga
cacttttcggtgagtcagccggcgccgtttcagtttcactgcacttactgtctccactgagtagaaacctttttaatcaagccatc
atggaatcaggatcctcaacagcaccttgggcaattttatcaagagaagagagttatagtagaggacttaggctggcaagg
gcaatgggttgtccagatgacagaaacgaaatacataaaacggtcgagtgcttaaggaaggcgaatagttcaacaatggtc
gagaaagaatgggaccacgtggctatatgtttttcccgtttgtcccggtggtggatggcgcttttcttgacgattatcctcaaa
aatcgctgtcaacaaacaattttaaaaaaacgaatatactcatgggtagtaactccgaagagggttactattcaatattttattat
ttgacggagcttttcaaaaaggaggaaaacgtggtggtgtctcgtgaaaattttgttaaagctattggacaacttaatccgaac
gcagatgcggcggttaaatcggctatagagtttgaatacactgattggttcagcccaaatgacccagaaaaaaatcgaaatg
ctctggacaaaatggtcggcgactatcagtttacatgcaacgtcaatgaattcgcacataaatatgcacttactggaaacaac
gtgtacatgtattattttaaacatcgttctttaaacaatccatggccgaaatggacaggggtgatgcacggtgatgaaatcagtt
atgtatttggagatcctttaaatccaaataaacgctacgaaatcgaagaaatcgaacttagcaagaaaatgatgagatactgg
accaattttgcaaaaacaggaaatccaagcaaaacatttgaagggtcttgggtcacgcccaagtggcccgtacacacggc
gtatggaaaagagtttctaacattagatacaaataacacttctatcggcgttgggccaagactagaacaatgtgctttttggaa
aaactacgttcctgatcttatggccatttcaaagagtatgaagtctgacaaaaactgtacaaccataagtggagggaccaaaa
cttacatgattgagttatcgctttggacaattgtgatgacaactgccgttttgatgTTAtgaatttcgttttaattcgtcttaaaca
aaaaaaaaa-3′
