# Supplementary figures and images for: Plant-Generated Artificial Small RNAs Mediated Aphid Resistance
Source: PLoS One. 2014 May 12;9(5):e97410. doi: 10.1371/journal.pone.0097410 (PMC4018293; doi:10.1371/journal.pone.0097410)

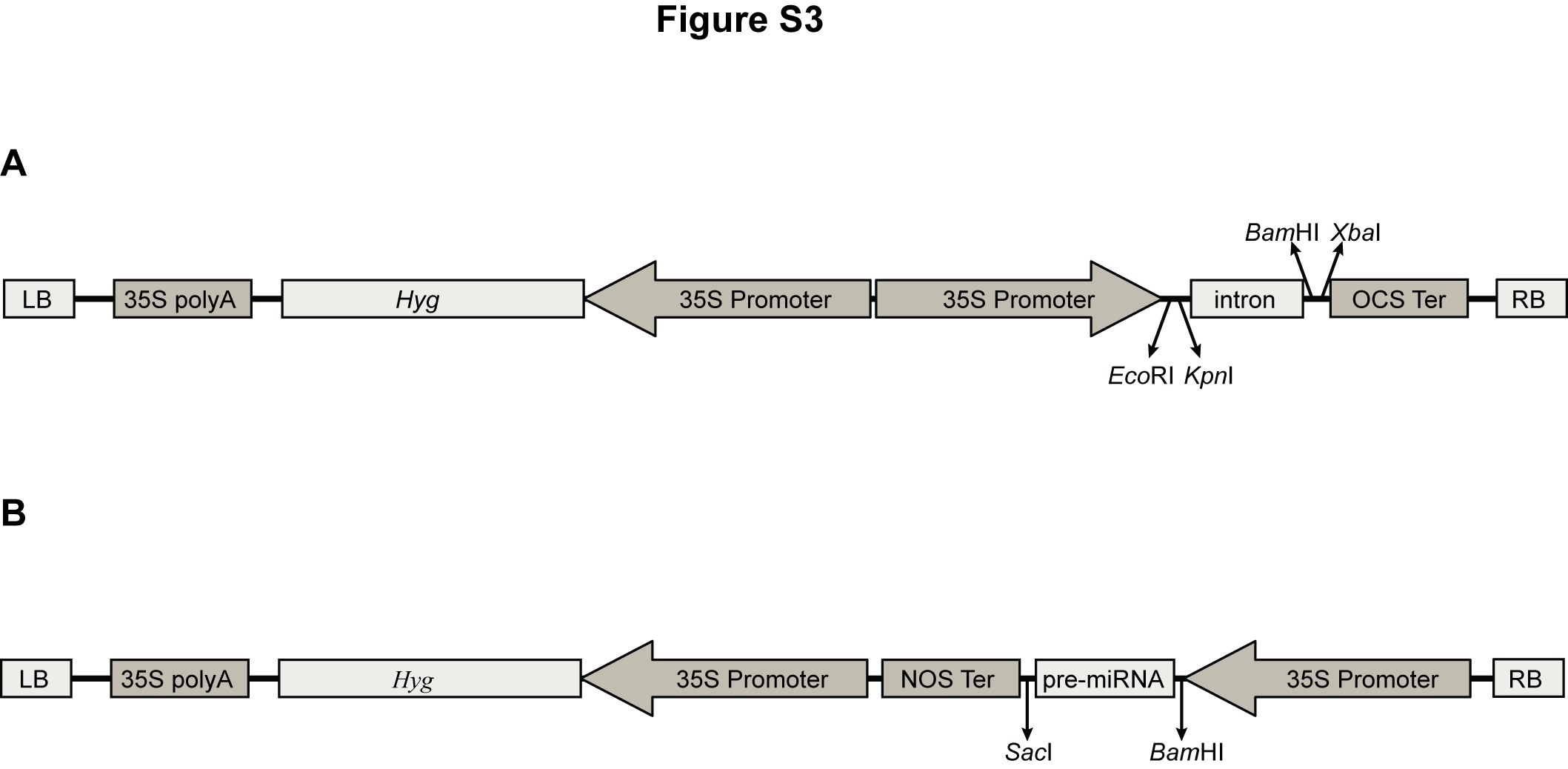

Supplement: Figure S3 — Structures of the constructs for expressing artificial hairpin RNAs (A) and microRNAs (B) in plants. (TIF) [file pone.0097410.s003.tif]
