# Supplementary material for: Plant-Generated Artificial Small RNAs Mediated Aphid Resistance
Source: PLoS One. 2014 May 12;9(5):e97410. doi: 10.1371/journal.pone.0097410 (PMC4018293; doi:10.1371/journal.pone.0097410)
Supplement: Tables S1 — List of primers used. (DOC) [file pone.0097410.s004.doc]

**Table S1. List of primers used**

| Gene | primers | sequence |
| --- | --- | --- |
| V-ATPaseE | A-EcoRI-F | 5'-AAAGAATTCAGCGACTCAAGATCATGGAGTAT-3' |
|  | A-KpnI-R | 5'-AAAGGTACCCTGCCACTTCTTCAACTAATTCT-3' |
|  | A-XbaI-F | 5'-AAATCTAGAAGCGACTCAAGATCATGGAGTAT-3' |
|  | A-BamHI-R | 5'-AAAGGATCCCTGCCACTTCTTCAACTAATTCT-3' |
| CPRR1 | B-EcoRI-F | 5'-AAAGAATTCCGTCGTTGCTGTTAGCTGTCGTC-3' |
|  | B-KpnI-R | 5'-AAAGGTACCGTGCTGGGCAGAGAGGCGAGGTA-3' |
|  | B-XbaI-F | 5'-AAATCTAGACGTCGTTGCTGTTAGCTGTCGTC-3' |
|  | B-BamHI-R | 5'-AAAGGATCCGTGCTGGGCAGAGAGGCGAGGTA-3' |
| Rps5 | C-EcoRI-F | 5'-AAAGAATTCGGGGAGATGTGGGAGCTACAATG-3' |
|  | C-KpnI-R | 5'-AAAGGTACCGTGTGAGTCTCTCTGGGTCCACT-3' |
|  | C-XbaI-F | 5'-AAATCTAGAGGGGAGATGTGGGAGCTACAATG-3' |
|  | C-BamHI-R | 5'-AAAGGATCCGTGTGAGTCTCTCTGGGTCCACT-3' |
| SMARCD1 | D-EcoRI-F | 5'-AAAGAATTCGACCCGCCGATAATCGAAATCCA-3' |
|  | D-KpnI-R | 5'-AAAGGTACCTGCGGTCCGATGCCATTCAACCA- 3' |
|  | D-XbaI-F | 5'-AAATCTAGAGACCCGCCGATAATCGAAATCCA-3' |
|  | D-BamHI-R | 5'-AAAGGATCCTGCGGTCCGATGCCATTCAACCA-3' |
| TBCD | E-EcoRI-F | 5'-AAAGAATTCTGGCATGGAGGATGTTTAGCACT-3' |
|  | E-KpnI-R | 5'-AAAGGTACCTGATGTGTCCCAATGTACTATTT-3' |
|  | E-XbaI--F | 5'-AAATCTAGATGGCATGGAGGATGTTTAGCACT-3' |
|  | E-BamHI-R | 5'-AAAGGATCCTGATGTGTCCCAATGTACTATTT-3' |
| delta-COP | F-EcoRI-F | 5'-AAAGAATTCCCGTAAGAACTGTATTATCGACT-3' |
|  | F-KpnI-R | 5'-AAAGGTACCTTAACACTCTCCCTATATCCAAG-3' |
|  | F-XbaI-F | 5'-AAATCTAGACCGTAAGAACTGTATTATCGACT-3' |
|  | F-BamHI-R | 5'-AAAGGATCCTTAACACTCTCCCTATATCCAAG-3' |
| Rps14 | J-EcoRI-F | 5'-AAAGAATTCAAGGAAGAGCCAGTAGTCCAACT-3' |
|  | J-KpnI-R | 5'-AAAGGTACCTACAAACGTCTACCTCTGCGACC-3' |
|  | J-XbaI-F | 5'-AAATCTAGAAAGGAAGAGCCAGTAGTCCAACT-3' |
|  | J-BamHI-R | 5'-AAAGGATCCTACAAACGTCTACCTCTGCGACC-3' |
| Med31 | K-EcoRI-F | 5'-AAAGAATTCGCTTATACCTATGACGTTTCACC-3' |
|  | K-KpnI-R | 5'-AAAGGTACCCTGATCATCATAAATTTCGAACA-3' |
|  | K-XbaI-F | 5'-AAATCTAGAGCTTATACCTATGACGTTTCACC-3' |
|  | K-BamHI-R | 5'-AAAGGATCCCTGATCATCATAAATTTCGAACA-3' |
| AChE2 | I-EcoRI-F | 5'-ACAATGTGGAATCCGAATTC-3' |
|  | I-KpnI-R | 5'-AAAAGGTACCAGACAGTAAGTGCAGTGAAA-3' |
|  | I-XbaI-F | 5'-AAAATCTAGAGAATTCCCCGGTATCTGAA-3' |
|  | I-BamHI-R | 5'-AAAAGGATCCAGACAGTAAGTGCAGTGAAA-3' |
|  | pre-miR171a/AchE2-1-F | 5'-CGGGATCCATGAGAGAGTCCCTTTGGAGCTTTTAAAAAAGGCGGAGATCTTACCTGACCACACACG-3' |
|  | pre-miR171a/AchE2-1-R | 5'-AAAAGAGCTCACGAGAGAGTACTGAGGAGCTTTTCAAAAAGGAGGAGATAATCTAGAGAGAATAATG-3' |
|  | pre-miR171a/AchE2-2-F | 5'-CGGGATCCATGAGAGAGTCCCTTTGTTTGGACGTTTGTGATTACAGATCTTACCTGACCACACACG-3' |
|  | pre-miR171a/AchE2-2-R | 5'-AAAAGAGCTCACGAGAGAGTACTGAGTTTGGACGATTGTGATGACAGATAATCTAGAGAGAATAATG-3' |
|  | AChE2-outer | 5'-CACTTACTGTCTCCACTGA-3' |
|  | AChE2-inner | 5'-GTTATAGTAGAGGACTTAGG-3' |
|  | poly(T) adapter | 5'-GCGAGCACAGAATTAATACGACTCACTATAGG(T)_12_VN-3' |
|  | Reverse primer | 5'-GCGAGCACAGAATTAATACGAC-3' |
|  | amiR-AChE2-1A | 5'-TCCTCCTTTTTGAAAAGCTCCA-3' |
|  | amiR-AChE2-2 | 5'-TGTCATCACAATCGTCCAAAC-3' |
|  | XT5.8S | 5'-ACGTCTGCCTGGGCGTCA-3' |
|  | AChE RT-F | 5'-GTTATAGTAGAGGACTTAGG-3' |
|  | AChE RT-R | 5'-GAGGATAATCGTCAAGAA-3' |
|  | Mpactin RT-F | 5'-ATGAAGTGCGATGTTGAC-3' |
|  | Mpactin RT-R | 5'-ATCTTGATTGTGCTTGGAG-3' |
